# Functional measurements based on feature tracking of cine magnetic resonance images identify left ventricular segments with myocardial scar

**DOI:** 10.1186/1476-7120-7-53

**Published:** 2009-11-16

**Authors:** Eva Maret, Tim Todt, Lars Brudin, Eva Nylander, Eva Swahn, Jan L Ohlsson, Jan E Engvall

**Affiliations:** 1 Department of Clinical Physiology, Ryhov County Hospital, SE-55185 Jonkoping, Sweden; 2 Center for Medical Image Science and Visualization, Linkoping University, SE-58185 Linkoping, Sweden; 3 Department of Medical and Health Sciences, Division of Cardiovascular Medicine, Linkoping University Hospital, SE-581 85 Linkoping, Sweden; 4 Department of Clinical Physiology, Kalmar County Hospital SE-39185 Kalmar, Sweden; 5 Department of Medical and Health Sciences/CVM/Clinical Physiology, Linkoping University Hospital, SE-581 85 Linkoping, Sweden

## Abstract

**Background:**

The aim of the study was to perform a feature tracking analysis on cine magnetic resonance (MR) images to elucidate if functional measurements of the motion of the left ventricular wall may detect scar defined with gadolinium enhanced MR.

Myocardial contraction can be measured in terms of the velocity, displacement and local deformation (strain) of a particular myocardial segment. Contraction of the myocardial wall will be reduced in the presence of scar and as a consequence of reduced myocardial blood flow.

**Methods:**

Thirty patients (3 women and 27 men) were selected based on the presence or absence of extensive scar in the anteroseptal area of the left ventricle. The patients were investigated in stable clinical condition, 4-8 weeks post ST-elevation myocardial infarction treated with percutaneous coronary intervention. Seventeen had a scar area >75% in at least one anteroseptal segment (scar) and thirteen had scar area <1% (non-scar). Velocity, displacement and strain were calculated in the longitudinal direction, tangential to the endocardial outline, and in the radial direction, perpendicular to the tangent.

**Results:**

In the scar patients, segments with scar showed lower functional measurements than remote segments. Radial measurements of velocity, displacement and strain performed better in terms of receiver-operator-characteristic curves (ROC) than the corresponding longitudinal measurements. The best area-under-curve was for radial strain, 0.89, where a cut-off value of 38.8% had 80% sensitivity and 86% specificity for the detection of a segment with scar area >50%. As a percentage of the mean, intraobserver variability was 16-14-26% for radial measurements of displacement-velocity-strain and corresponding interobserver variability was 13-12-18%.

**Conclusion:**

Feature tracking analysis of cine-MR displays velocity, displacement and strain in the radial and longitudinal direction and may be used for the detection of transmural scar. The accuracy and repeatability of the radial functional measurements is satisfactory and global measures agree.

## Background

Wall motion abnormalities (WMA) of the left ventricle are often caused by coronary artery disease, most frequently the result of a myocardial infarction (scar), or from acute (stunning) as well as chronic (hibernation) reduction in segmental blood flow. WMA can be detected by visual inspection, which is fast but dependent on the experience of the operator [1], or by more objective means such as measuring myocardial velocity [2], deformation [3,4] or using myocardial tagging [4-6]. Unless compensated for by hyperkinetic wall motion in remote areas, the physiological effect of a WMA is a reduction in the left ventricular ejection fraction (LVEF) which is closely related to prognosis in chronic coronary artery disease [7-11]. The size of the myocardial scar is another strong prognostic parameter that is best determined with late gadolinium enhancement (LGE) imaging using magnetic resonance (MR), [12].

2D-strain has been pioneered in echocardiography and advocated for its angle independence in contrast to strain determined with tissue Doppler. The determination of 2D-strain utilizes the presence of natural ultrasound reflectors in the myocardium, "speckle". The in-plane motion of these reflectors can be tracked through the cardiac cycle and the varying distances between speckles utilized for calculating 2D-strain. Balanced steady state free precession turbo field-echo (B-SSFP TFE) cine-MR images contain less variation in myocardial tissue signals than seen in echocardiographic images but still allow tracking of mechanical deformation. The aim of this study was to apply a new feature tracking software (Diogenes MRI, Tomtec GmbH, Unterschliessheim, Germany) on cine-MR images to evaluate its utility and ability to measure velocity, displacement and deformation and thereby detect the segmental distribution of infarcted myocardium.

## Methods

### Study population

The study population was selected from 99 patients included in a study of primary PCI for ST-elevation myocardial infarction (STEMI). These patients were recruited between February 2006 and September 2007 and agreed to return for infarct size determination with MRI 6 ± 2 weeks after primary PCI. Thirty patients (3 women and 27 men, age 62 ± 11 years, height 177 ± 7 cm, weight 85 ± 11 kg) were selected based on the presence or absence of extensive myocardial scar in anterior and anteroseptal segments that are considered to belong to the LAD territory [13]. Seventeen patients with scar extent >75% in at least one segment belonging to this area (scar patients) and thirteen without scar in this area or in any other parts of the myocardium were selected (non-scar patients, i.e. some patients did not develop chronic scar despite presenting with unequivocal signs of STEMI). The intention was to contrast the possible effects of a scar on the functional parameters that were to be determined with the feature tracking software. The anteroseptal area was selected because it was the most frequent location of myocardial damage in this study population. One scar patient had his culprit lesion in the right coronary artery (RCA), all others in the LAD-system. In the non-scar group, the lesion treated with PCI was within the LAD/diagonal system in 7 patients and within the RCA/left circumflex coronary artery (LCX) system in 6 patients. Additional stenoses not dilated at the index event were seen in other coronary arteries in 7 of the 17 scar patients and in two of the 13 non-scar patients. Three patients in the scar group had a history of previous myocardial infarction. Two of these and one patient in the non-scar group had undergone PCI. None of the patients had been subjected to coronary by-pass surgery. Initial exclusion criteria were unwillingness to participate in the study or those related to performing MRI such as pacemaker, atrial fibrillation or claustrophobia.

The study complied with the Declaration of Helsinki and with agreements on Good Clinical Practice. Approval was obtained by the Regional Ethical Review Board in Linköping. Written informed consent was obtained from all study participants.

### MR imaging

MR exams were performed on a Philips 1.5T Achieva scanner (Philips Healthcare, Best, the Netherlands). A five-element cardiac synergy surface coil was used for signal reception in all measurements. ECG-triggered MR images were obtained during repeated breath-holds. Cine-MR was performed with a B-SSFP TFE sequence and attempted to cover the entire left ventricle with on average 19 (range 17-25) short-axis slices and three long axis planes (2- and 4- chamber views as well as the apical long-axis view). Slice thickness was 10 mm and slice gap -5 mm (i.e. slices were overcontiguous). Temporal resolution ranged between 26-41 ms (30 acquired phases). The contrast-enhanced images were acquired at the same slice positions as the cine-images, about 20 min after the administration of gadopentate dimeglumine (Gd-DTPA) 0.2 mmol/kg bodyweight (Schering Nordiska AB, Järfälla, Sweden). The inversion recovery turbo field echo (IR-TFE) sequence was a segmented 3D spoiled gradient echo sequence with TE = 1.3 ms, TR = 4.4 ms and TFE factor 43, leading to an acquisition phase time of 188 ms acquired during diastole. The inversion time was on average 270 ms (range 225-340 ms).

### Left ventricular size and function

Left ventricular end-diastolic and end-systolic volumes as well as ejection fraction were determined from the cine short axis loops on a stand-alone workstation (View Forum R6.3, Philips Electronics, Eindhoven, the Netherlands, http://www.healthcare.philips.com/main/about/Connectivity/dicom_statements/workstations_statements.wpd) while analysis of velocity, displacement and strain was performed with the feature tracking software (Diogenes MRI, Tomtec GmbH - related information regarding the echocardiographic version of the software can be found at http://www.tomtec.de/end_users/2d_echo/cardiac_performance_analysisc.html) using a standard laptop computer. The apical 2- and 4-chamber views as well as the apical longaxis view were used, after conversion of the DICOM image stack to avi-files.

### Infarct size and transmurality

**Figure 1:**
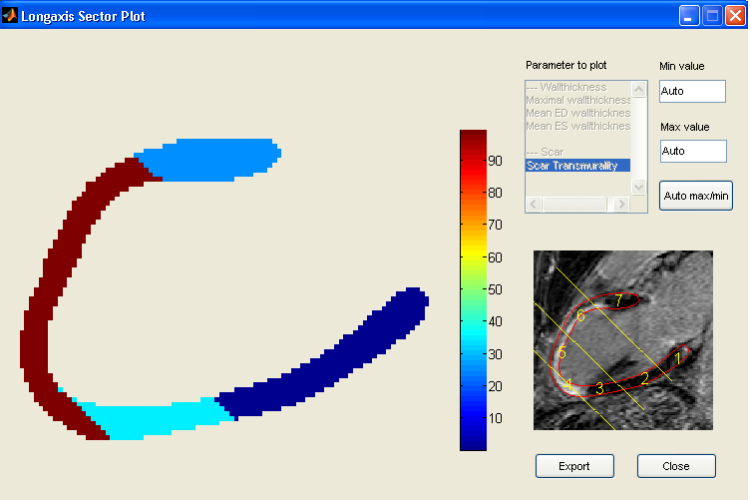
**Transmurality of scar calculated from LGE image in the 2-chamber view**. Scar is 100% transmural along the middle and apical part of the anterior wall extending to the apical part of the inferior wall. Calculation performed with the Segment software.

Myocardial scar was visualized with the late gadolinium enhancement technique. Infarct size was determined in millilitres and as a percentage of left ventricular mass from the stack of shortaxis images, using a computer freeware "Segment", http://segment.heiberg.se. The infarct segmental scar area was determined in 3 apical views using "Segment" (Figure 1). Transmurality was in this setting defined as segmental scar area, i.e. infarct area divided by segment area. A scar segment was defined as any segment with scar ≥1% (small areas of enhanced signal may be caused by imperfect segmentation of e.g. the ventricular cavity and is considered to constitute noise). Adjacent segments were those in the LAD territory without scar. Remote segments were those in the territory of the right (RCA) and circumflex (LCX) coronary arteries. Interobserver variability for the determination of scar volume with "Segment" for our group has been reported as 4.2 ml [14].

### Feature tracking analysis

**Figure 2:**
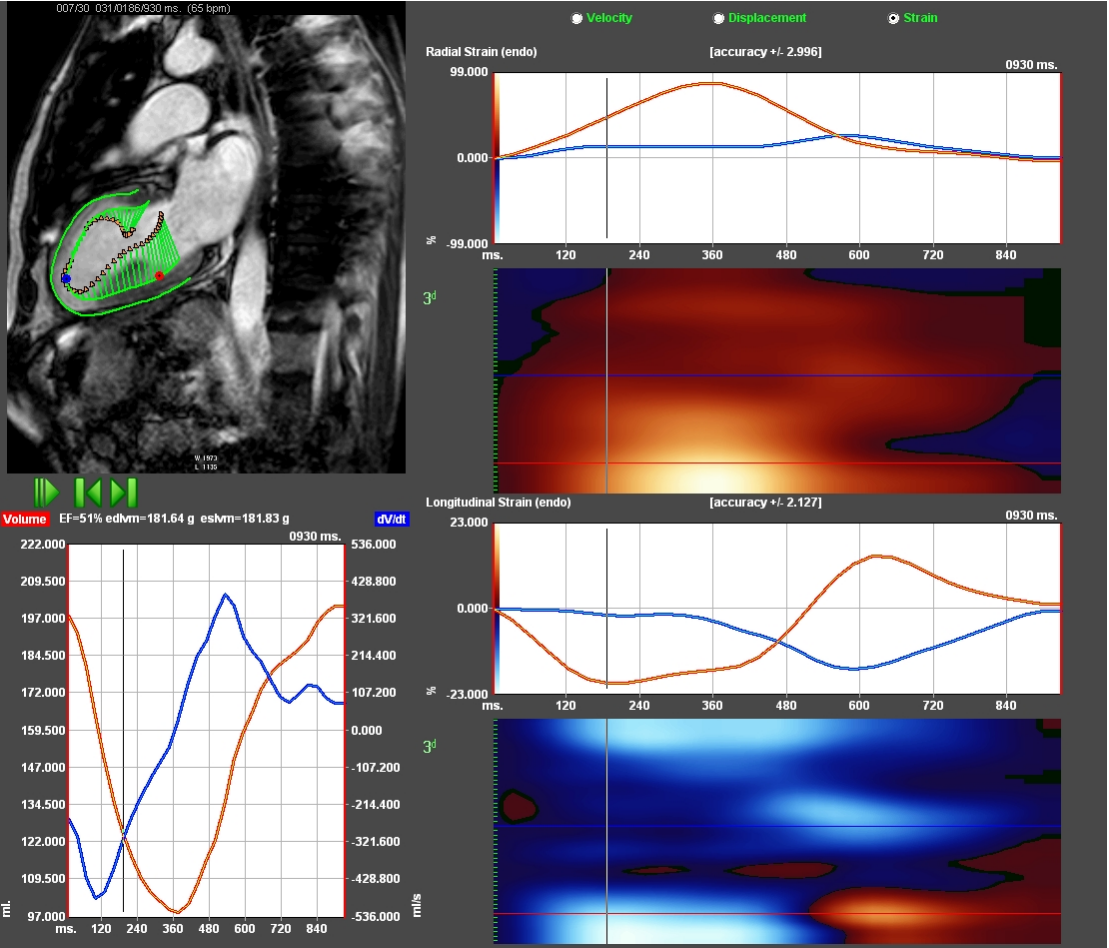
**Feature tracking of patient with extensive scar as shown in Figure 1**. Upper left shows vector arrows of late systolic velocity tracing, 190 ms after QRS (green). Upper right shows radial strain tracing of the entire cardiac cycle. Blue represents the apex showing very low strain values; red is the normal posterior wall. Lower right displays longitudinal traces from the same locations, with postsystolic strain in the apex. Lower left is volume curve based on single plane Simpson (red) and emptying velocity (dV/dt), blue tracing.

**Figure 3:**
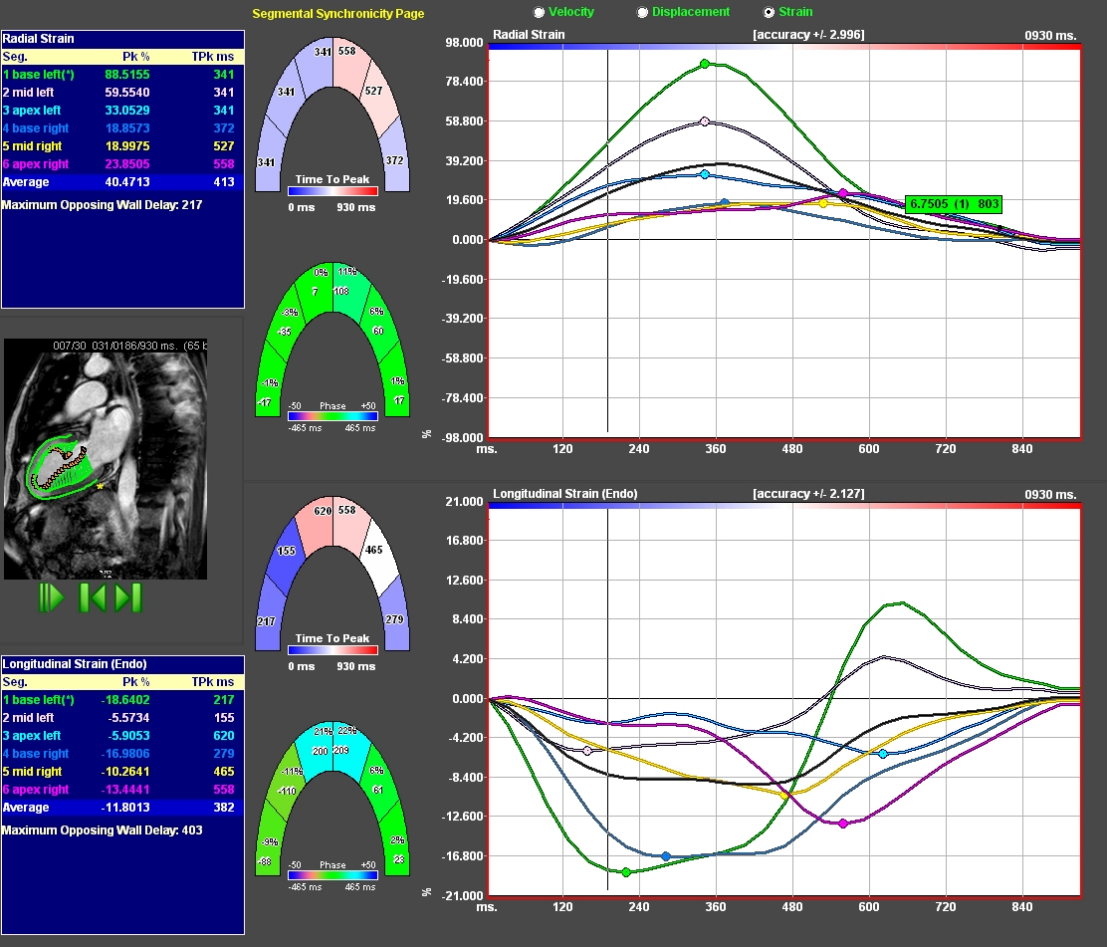
**Measurement window of feature tracking software**. Middle figures show graphical display of 6 segments, three anterior and three inferoposterior. Right upper panel shows radial strain tracings from the six segments. Lower right panel depicts the corresponding longitudinal strain values. Velocity and displacement can be selected for alternative presentation. Left blue box shows peak values and time to peak for corresponding segments.

After manually delineating the endocardium and epicardium in diastole, the software tracked the motion of the wall through the entire cardiac cycle (additional file 1, 2). Velocity, displacement and strain were calculated in 48 points (tangential to the endocardial outline, assumed positive in the base-to-apex direction), and in the radial direction (perpendicular to the tangent, positive inward; Figure 2). The left ventricular wall was divided into 6 segments in each of the three views (in pairs at each level, base-mid-apical), giving a total of 18 segments (Figure 3). For the definition of coronary territories, the apical anteroseptal and inferoseptal segments were assigned to the apical septal segment and the anterolateral and inferolateral segments were assigned to the apical lateral segment of the AHA model. The highest systolic value in each segment was used regardless of the presence of a higher postsystolic peak. The tracing of the myocardium was repeated three times and the mean value of the functional measurements was used. In addition, the software calculated a mean value for the six segments in each view and for each parameter. A single-plane left ventricular ejection fraction was reported in each view and an average was calculated from the apical 2- and 4-chamber views for each patient. Global functional measures were calculated for all parameters as an average value from the three apical views, including all 18 segments.

### Statistical analysis

Statistical analyses were performed using SPSS 16.0 (SPSS Inc., Chicago, Illinois, USA) as well as Statistica 8.0 (Statsoft Inc, Tulsa, Oklahoma, USA). All variables were reasonably well normally distributed why parametric tests were used. Paired and unpaired 2-tailed Student's t-tests were used along with ANOVA (followed by Duncan's test in case of significance) and Pearson correlation coefficient when appropriate. Receiver-operator-characteristics (ROC) curve analyses were performed using the statistical programme MedCalc^® ^Version 6.10 (MedCalc Software, Mariakerke, Belgium). Intra-and interobserver variability of the functional measures was expressed as standard error of a single determination (S_method_) using the formula first proposed by Dahlberg [15]. S_method _was also expressed as % over all means. Single measure intraclass correlation coefficient (ICC) was also used to express interobserver variability. ICC assesses rating reliability by comparing the variability of different ratings of the same subject with the total variation across all ratings and all subjects [16,17].

## Results

### Left ventricular volumes and LVEF

In the scar patients, scar size was on average 31 ± 12 ml or 17 ± 8% of the left ventricular myocardium. Twelve of the 17 patients had a scar percentage exceeding 12% which is considered prognostically unfavourable [18]. Left ventricular end-diastolic volume (LVEDV) and end-systolic volume (LVESV) were significantly larger and left ventricular ejection fraction (LVEF) lower in the scar group compared with the non-scar group (Table 1). LVEDV measured on cine-MRI (View Forum) as well as on LGE still images (Segment), was160 ± 42 ml vs. 157 ± 40 ml in the scar group, and 137 ± 16 ml vs. 128 ± 27 ml in the non-scar group (p = 0.80 and p = 0.30 respectively). LVEF measured on shortaxis cine-MR, compared with biplane feature tracking, showed no significant differences in the non-scar group (60 ± 8% vs. 64 ± 4%, p = 0.07) but was lower with cine-MR than with feature tracking in the scar group (39 ± 9% vs. 50 ± 8%, p = 0.001).

**Table 1:** Global values of volumes and mass of the left ventricle.

	Scar	Non-scar	p
	(n = 17)	(n = 13)	
**Cine-MRI**			
**View Forum**			
LVEDV, ml	160 ± 42 (102 - 269)	137 ± 16 (115 - 170)	0.049
LVESV, ml	99 ± 35 (51 - 188)	55 ± 16 (25 - 90)	0.0001
LVEF,%	39 ± 9 (19 - 53)	60 ± 8 (47 - 78)	<0.0001
**Diogenes MRI**			
LVEF,%	50 ± 8 (31-64)	65 ± 4 (58-70)	<0.0001
			
**LGE-MRI**			
**Segment**			
LVEDV, ml	157 ± 40 (113 - 264)	128 ± 27 (81 - 166)	0.027
LVmass, g	190 ± 37 (151 - 266)	154 ± 25 (110 - 180)	0.004
LVscar,%	16.6 ± 8.4 (1 - 35)	0.2 ± 0.4 (0 - 1)	-
Transmurality	number of segments	number of segments	
<1%	141	234	
1-25%	36	0	
26-50%	22	0	
51-75%	29	0	
76-100%	78	0	

Mean ± SD (range)

### Segmental scar area

In the scar group, scar area was 52 ± 39% in the anteroseptal segments and 4 ± 18% in the remote segments. In one of the patients, the segment with >75% scar was located only in the apical cap. Eleven out of 119 remote segments showed small scar areas probably due to slight imperfections in the segmentation (noise) or due to the vascular supply being different from the standard segment model. Significant gadolinium uptake was not seen in the non-scar group.

### Functional measurements

**Figure 4:**
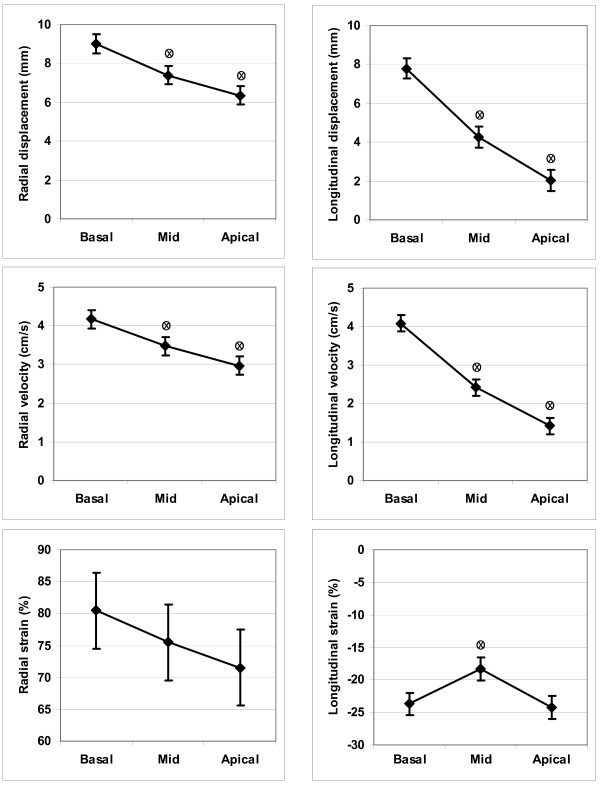
**Functional measures vs. location, non-scar patients**. Left sided panels show radial displacement (upper), velocity (mid) and strain (lower), from three different segment levels, apex-mid-base. Right sided panels show the corresponding longitudinal values. o = denotes statistically significant difference (p < 0.05) compared to basal x = denotes statistically significant difference (p < 0.05) compared to nearest left value

**Figure 5:**
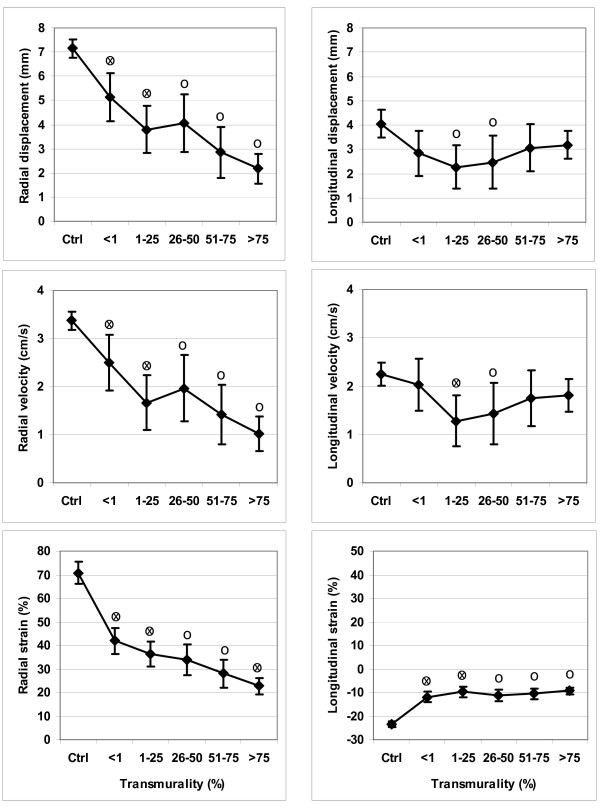
**Functional measures vs. transmurality, all patients (Ctrl = non-scar)**. Left-sided panels show radial displacement (upper), velocity (mid) and strain (lower) in segments with various degree of transmurality. Right-sided panels show the corresponding longitudinal values. o = denotes statistically significant difference (p < 0.05) compared to controls (Ctrl) x = denotes statistically significant difference (p < 0.05) compared to nearest left value

Results from the feature tracking analysis of the non-scar patients are reported in Figure 4. Radial strain did not show significant differences between the basal, mid and apical segments. Analysis of radial and longitudinal displacement, radial and longitudinal velocity and longitudinal strain showed, on the contrary, significant differences between the segment levels. Results of the functional measurements for the anteroseptal segments in the scar group compared with the corresponding segments in the non-scar group, stratified for scar segment area, are reported in Figure 5. In summary, for segments with a scar area 51-75%, as well as for segments with a scar area >75, significant differences were seen between scar and non-scar for all radial measures and for longitudinal strain. Longitudinal velocity and displacement showed less difference, possibly due to difficulties in longitudinal tracking by the software.

In the scar patients, anteroseptal segments showed, as expected, lower functional measurements than remote segments, Table 2. The remote segments in the scar group showed in turn lower functional measurements than the remote segments in the non-scar group. In the non-scar patients, "remote" segments showed higher velocity, displacement and strain than anteroseptal segments, in line with previous findings [11].

**Table 2:** Functional measurements for LAD-segments vs. remote segments in all patients.

	Scar				Non-scar			
		
	LAD (n = 187)		Remote (n = 119)		LAD (n = 187)		Remote (n = 119)	
				
	Mean	(95% CI)	Mean	(95% CI)	Mean	(95% CI)	Mean	(95% CI)
**Displacement, mm**								
Radial	3.28*	(2.85-3.72)	7.09^§^	(6.51-7.68)	7.15*	(6.76-7.53)	8.19	(7.65-8.73)
Longitudinal	2.87*	(2.51-3.23)	4.10^§^	(3.57-4.64)	4.05*	(3.48-4.62)	5.77	(5.03-6.50)
								
**Velocity, cm/s**								
Radial	1.56*	(1.31-1.80)	3.32^§^	(3.06-3.57)	3.37*	(3.17-3.57)	3.74	(3.49-3.99)
Longitudinal	1.71*	(1.50-1.92)	2.62^§^	(2.30-2.94)	2.25*	(2.01-2.48)	3.26	(2.94-3.57)
								
**Strain, %**								
Radial	30.50*	(27.60-33.39)	65.90^§^	(60.80-70.99)	70.80*	(66.24-75.37)	80.49	(72.20-88.78)
Longitudinal	-10.3*	(-11.2 to -9.3)	-15.8^§^	(-17.4 to -14.2)	-23.3*	(-24.5 to -22.1)	-19.9	(-21.9 to -17.9)

Significance testing for LAD-area vs. remote area for the two categories, * = p < 0.05

Significance testing for the remote areas for the two categories, § = p < 0.05

**Figure 6:**
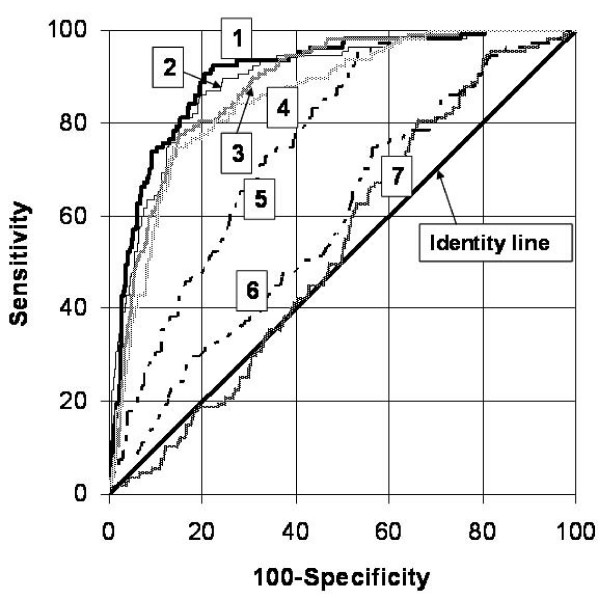
**ROC curves for the functional measures vs. >50% transmurality**. The composite of the radial measures strain-displacement-velocity shows only marginally larger AUC than radial strain alone. ROC curves for the detection of 50% segmental scar using 1 = Composite, 2 = Radial strain, 3 = Radial displacement, 4 = Radial velocity, 5 = Longitudinal strain, 6 = Longitudinal velocity, 7 = Longitudinal displacement

Receiver-operator-characteristics curves (ROC) were constructed for all measurements (Figure 6, Table 3 and Table 4). They were primarily used to distinguish segments with scar area >50% as well as >75% vs. non-transmural scar. The area-under-curve for all measures vs. >50% scar area are shown in table 3, where also sensitivity and specificity for different cut-off levels are given. The best area under curve (AUC) was for radial strain, where a cut-off value of <38.8% detected a segment with scar area >50% among anteroseptal segments with 80% sensitivity and 86% specificity. A longitudinal strain cut-off of -18.5% had an AUC-value of 0.76 for the detection of a scar area >50% among anteroseptal segments (sensitivity 47% and specificity 95%).

**Table 3:** Areas under the receiver operating characteristic (ROC) curves (AUC) shown in Figure 3.

	Composite	Radial strain	Radial displacement	Radial velocity	Longitudinal strain	Longitudinal velocity	Longitudinal displacement
**ROC area**	0.905	0.892	0.879	0.855	0.764	0.588	0.535
**95% conf. int**.	0.877-0.929	0.863-0.917	0.848-0.906	0.822-0.883	0.726-0.799	0.545-0.630	0.491-0.578
Cut off value*	2.52	38.8	3.97	1.82	-18.5	2.49	4.99
**Sens/Spec**	78/93	80/86	85/78	86/75	47/95	44/75	34/80
Cut off value*	>2.4	>38.9	>4.47	>2.1	<-9.6	>1.1	>1.29
**Sens at Spec = 80%**	90	86	80	78	48	31	19

* Chosen cut off value corresponding to sensitivity and specificity in the row below.

The parameters are ranked after AUC. Sensitivities (sens) and specificities (spec) are in %. Significance of differences between ROC areas and sensitivities at 80% specificity are shown in Table 4.

**Table 4:** P-values from comparisons of ROC Areas and sensitivities at 80% specificity

	Radial strain	Radial displacement	Radial velocity	Longitudinal strain	Longitudinal velocity	Longitudinal displacement
**Composite**	0.160 (0.457)	0.001 (0.072)	<0.001 (0.093)	<0.001 (<0.001)	<0.001 (<0.001)	<0.001 (<0.001)
**Radial strain**	-	0.302 (0.567)	0.015 (0.417)	<0.001 (<0.001)	<0.001 (<0.001)	<0.001 (<0.001)
**Radial displacement**		-	0.011 (0.780)	<0.001 (0.004)	<0.001 (<0.001)	<0.001 (<0.001)
**Radial velocity**			-	0.001 (0.012)	<0.001 (<0.001)	<0.001 (<0.001)
**Longitudinal strain**				-	<0.001 (0.227)	<0.001 (0.021)
**Longitudinal velocity**					-	<0.001 (0.114)

P-values for differences between the ROC areas and, within parenthesis, for sensitivities at 80% specificity using an exact statistical method corresponding to McNemar's paired Chi2 modification.

**Figure 7:**
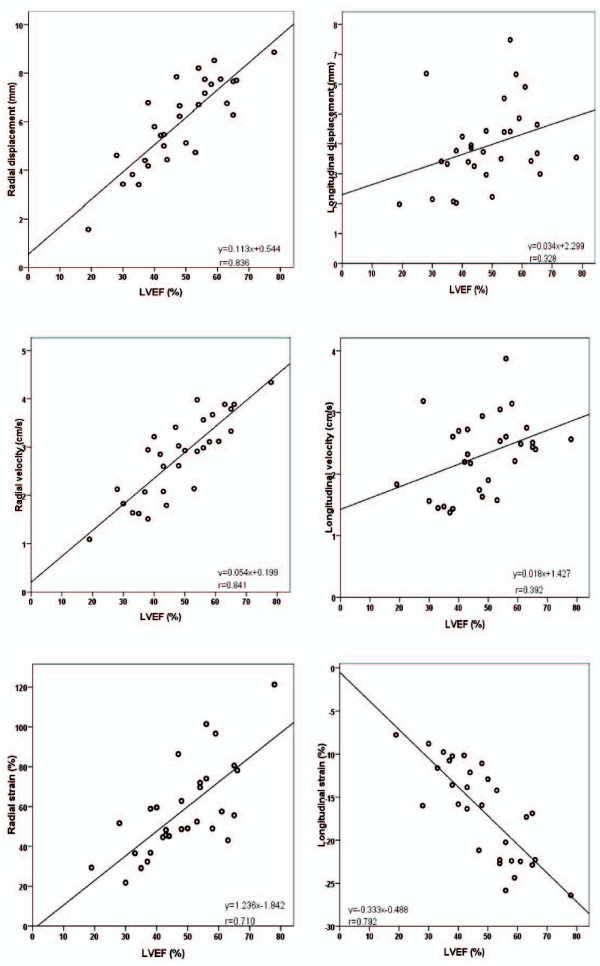
**Correlation between global functional measurements and MR-determined LVEF**. Highest correlation (r = 0.84, 0.84 and 0.79) was between radial displacement, radial velocity and longitudinal strain vs. ejection fraction determined from a stack of shortaxis cine MRI.

The global functional measurements calculated from the apical cine images (mean of 18 segments per patient) compared with MR-determined LVEF are shown in Figure 7. The best correlation was seen between the radial measures and LVEF in addition to longitudinal strain and LVEF.

### Intra- and interobserver variability

Intraobserver variability was estimated from three repeated tracings on 5 patients in the scar and 5 patients in the non-scar group by two investigators one month after the original measurement (Table 5). As a percentage of the mean, intraobserver variability was 16-14-26% for radial measurements of displacement-velocity-strain and corresponding interobserver variability was 13-12-18%. ICC was better than 0.7 for all measurements (see table). ICC above 0.6 is considered good and excellent if >0.75 [19].

**Table 5:** Intra- and interobserver variability

				Intraobserver variability (one measurement)		Interobserver variability (mean of 3 measurements)	
	ICC	P	Median	Smethod (absolute)	Smethod (% of mean)	Smethod (absolute)	Smethod (% of mean)
**Displacement, mm**							
**Radial**	0.93	<0.001	7.1	**0.75**/0.74/0.75	**11**/16/8	**0.62**/0.61/0.64	**9**/13/7
**Longitudinal**	0.90	<0.001	3.4	**1.16**/0.83/1.41	**27**/49/20	**0.78**/0.47/0.99	**18**/28/14
							
**Velocity, cm/s**							
**Radial**	0.90	<0.001	3.4	**0.39**/0.33/0.43	**12**/14/10	**0.29**/0.28/0.29	**9**/12/7
**Longitudinal**	0.79	<0.001	2.3	**0.71**/0.46/0.90	**28**/33/24	**0.47**/0.27/0.61	**19**/19/17
							
**Strain, %**							
**Radial**	0.74	<0.001	55.4	**10.9**/9.0/12.5	**21**/26/18	**7.4**/6.5/8.2	**14**/18/12
**Longitudinal**	0.77	<0.001	-17.0	**4.6**/3.5/5.4	**26**/33/22	**2.7**/2.5/2.9	**15**/23/12

Intraobserver variability expressed in terms of intraclass correlation coefficient and intra- and interobserver variability according to Dahlberg as absolute and relative values (**all values**/below median/above median).

## Discussion

### Main findings of the study

In this study we show that the feature-tracking software is able to track left ventricular wall motion with precision, as shown by low intra- and interobserver variability. Scarred anteroseptal myocardial segments can be differentiated from the corresponding non-scar segments using any of the three radial parameters, but most of the information is contained in the measurement of radial strain. We also show that a cut-off value of 38.8% radial strain identifies segments with >50% scar area. Measuring longitudinal motion was less successful, even if segments with and without scar may be differentiated with the aid of longitudinal strain, in line with previous reports using an earlier version of the software applied to echocardiographic images[20]. Also in line with previous findings [11,21], the global average of the radial measures correlated well with LVEF indicating that these measurements have a validity that can be corroborated by other parameters that are more frequently used. These findings indicate that much more functional information can be derived from cine-MRI than is usually collected in the follow-up of patients post myocardial infarction. We suggest that this information should be collected and analysed, facilitating an improved treatment of patients.

### Scar size and segmental scar area

MR-determined scar size from multiple short axis cut planes is exact and reproducible [12]. In many studies, the transmurality of the scar has been shown to determine the likelihood of improvement after revascularization [22]. Here, we defined transmurality as infarct area per segment from scar recordings in cut planes identical to the ones where the cine-loops were recorded, which ensures that the functional measurements refer to scar size as seen on the LGE images.

### Functional measurements

Feature tracking is a new approach for assessing myocardial motion. We could show that velocity, displacement and peak systolic strain in the radial direction were able to differentiate segments with varying extent of scar and that most of the information was present in the measurement of radial strain. In the longitudinal direction, the only measure that identified scarred segments was strain, in contrast to velocity and displacement. This could be explained by M-mode edge detection in the radial direction tracking motion superiorly compared with the longitudinal method, or possibly by strain being less influenced by the tethering effect of adjacent segments in the longitudinal direction. In contrast, Marwick [23] found that radial strain from velocity vector imaging (VVI) on echo did not correlate with radial strain from HARP analysis of tagging MRI. Differences in tissue contrast between ultrasound and MRI could be important in that situation. In non-scar patients, functional measurements in remote areas were higher than the same measurements in the anteroseptal area, possibly because anteroseptal segments in general are more apically located than remote segments, which is in line with previous findings [24]. Strain values are dependent on the shortening fraction of myocardial muscle fibres as well as on their main orientation in regard to the long- and shortaxis of the ventricle. Ventricular fibre orientation is complex [25,26]. Strain values, analysed with tagging MRI, have been shown to differ between the subendocardium and epicardium [27]. Newer MRI-based techniques such as fibre tracking have supported these findings [28]. However, we could not detect significant differences in radial strain between apex, mid and basal locations in the non-scar patients (Figure 4). Strain has been shown to be sensitive to effects of afterload as well as ventricular size, being less in larger ventricles [29]. This might explain some of the findings in this study, where radial strain was significantly lower in the remote segments of the larger scarred ventricles compared to non-scarred ventricles. However, these differences were small. Blood pressure, which could have influenced the functional measures [30], was not recorded at the MRI exam. The result of the PCI of the culprit lesion was deemed satisfactory in all cases. An unsatisfactory revascularization could possibly have caused hibernation of viable myocardium in the peri-infarct zone, accentuating the differences in functional parameters between scar and non-scar patients. This was considered unlikely since patients were in stable condition on average 6 weeks after the acute event. An additional explanation for differences between the remote segments of the scar vs. non-scar groups could have been differences in the extent of coronary disease, with more stenoses remaining in vessels supplying the remote area in the scar- compared with the non-scar patients. This was partly true in terms of 7 out of 17 patients in the scar group having significant stenoses in vessels other than the culprit lesion compared to 2 out of 13 patients in the non-scar group.

All functional measures could be recalculated to reflect the global impact of scarring on the left ventricles. Whether these measures can be used for prognostication, in line with the use of LVEF, requires further investigations in larger studies [11].

Should echocardiographic methods be abandoned for the determination of necrotic myocardium, in favour of MRI? Echo-determined wall thickness <5.5 mm suggests transmural scar [31]. Echo is less specific than MRI but is considerably cheaper (factor of four in our institution), is available at the bedside, and has no contraindications. Thus, in a clinical situation, echo still defends its role for hemodynamic as well as for functional assessment of myocardial infarction.

### Limitations

A larger cohort of patients is needed to test the behaviour of the software in patients with disease in all three coronary territories as well as a larger number of infarctions with subendocardial distribution. Note that values of sensitivity and specificity using suggested cut-off values always give an overoptimistic picture when first adopted. The definition of transmurality as segmental infarct area may be debatable but was considered more objective than the visual determination often used. We also acknowledge that the calculation of ejection fraction using biplane Simpson is less accurate than using a stack of shortaxis images but the biplane approach is recommended in echocardiographic guidelines [32] and is simpler to use. Finally, the potential development of restenosis between the initial PCI and the MRI exam at 6 weeks, which could have affected strain measurements, was only excluded on clinical ground.

## Conclusion

Feature tracking is able to detect progressively increasing segmental scar area ("transmurality") from a functional analysis of cine-MR. A cut-off value for radial strain of 38.8% detected a segment with scar area >50% within the LAD distribution with 80% sensitivity and 86% specificity. The accuracy and repeatability of the radial functional measurements are satisfactory and global measures agree with other aspects of global left ventricular function. Further studies are needed for determining the predictive value of this method in individual patients.

## Competing interests

The authors declare that they have no competing interests.

## Abbreviations

Anova: analysis of variance; b-SSFP TFE: balanced steady state free precession turbo field echo; DICOM: digital imaging and communications in medicine; ECG: elektrocardiogram; ICC: intraclass correlation coefficient; IR-TFE: inversion recovery turbo field echo; LAD: left anterior descending artery; LCX: left circumflex coronary artery; LGE: late gadolinium enhancement; LV: left ventricle; LVEF: left ventricular ejection fraction; LVEDV: left ventricular end-diastolic volume; LVESV: left ventricular end-systolic volume; MRI: magnetic resonance imaging; PCI: percutaneous coronary intervention; RCA: right coronary artery; ROC: receiver-operator-characteristics; SAX: short axis; SD: standard deviation; SPSS: statistical package for the social sciences; STEMI: ST-elevation myocardial infarction; TR: repetition time; WMA: wall motion abnormality

## Authors' contributions

EM planned the study, investigated some of the patients, performed measurements and analyses and took a major part of writing the manuscript. TT and ES participated in planning the study. LB participated in the statistical analyses of the results and in writing of the manuscript. EN participated in the writing of the manuscript. JO took part in statistical analyses and in writing of the manuscript. JE planned the study, investigated all the patients, performed measurements and analyses and took a major part in the writing of the manuscript. All authors have read and approved the final manuscript.

## Funding

This project was supported by Futurum - the academy for healthcare, Jönköping County Council, the Swedish Heart Lung Foundation, the Swedish Research Council, the Medical Research Council of Southeast Sweden and the Centre for Medical Image Science and Visualization, Linköping University Hospital.

## Supplementary Material

Additional file 1 **Vector presentation (Diogenes MRI) of endocardial motion tracking**. Cine-MRI, apical 2-chamber view of anterior myocardial infarction. Arrows show direction and velocity of endocardial motion. Click here for file

Additional file 2 **Tracking loops (Diogenes MRI) of endocardial motion**. Cine-MRI, apical 2-chamber view of anterior myocardial infarction. Click here for file
